# S 47445 Produces Antidepressant- and Anxiolytic-Like Effects through Neurogenesis Dependent and Independent Mechanisms

**DOI:** 10.3389/fphar.2017.00462

**Published:** 2017-07-19

**Authors:** Indira Mendez-David, Jean-Philippe Guilloux, Mariusz Papp, Laurent Tritschler, Elisabeth Mocaer, Alain M. Gardier, Sylvie Bretin, Denis J. David

**Affiliations:** ^1^CESP/UMRS-1178, Faculté de Pharmacie, Institut National de la Santé et de la Recherche Médicale, Université Paris-Sud – Université Paris-Saclay Chatenay-Malabry, France; ^2^Institute of Pharmacology, Polish Academy of Sciences Krakow, Poland; ^3^Institut de Recherches Internationales Servier Suresnes, France

**Keywords:** AMPAR, positive allosteric modulator, corticosterone, chronic mild stress, antidepressant, neurogenesis, BDNF, fluoxetine

## Abstract

Glutamatergic dysfunctions are observed in the pathophysiology of depression. The glutamatergic synapse as well as the AMPA receptor’s (AMPAR) activation may represent new potential targets for therapeutic intervention in the context of major depressive disorders. S 47445 is a novel AMPARs positive allosteric modulator (AMPA-PAM) possessing procognitive, neurotrophic properties and enhancing synaptic plasticity. Here, we investigated the antidepressant/anxiolytic-like effects of S 47445 in a mouse model of anxiety/depression based on chronic corticosterone administration (CORT) and in the Chronic Mild Stress (CMS) model in rats. Four doses of S 47445 (0.3 to 10 mg/kg, oral route, 4 and 5 weeks, respectively) were assessed in both models. In mouse, behavioral effects were tested in various anxiety-and depression-related behaviors : the elevated plus maze (EPM), open field (OF), splash test (ST), forced swim test (FST), tail suspension test (TST), fur coat state and novelty suppressed feeding (NSF) as well as on hippocampal neurogenesis and dendritic arborization in comparison to chronic fluoxetine treatment (18 mg/kg, p.o.). In rats, behavioral effects of S 47445 were monitored using sucrose consumption and compared to those of imipramine or venlafaxine (10 mg/kg, i.p.) during the whole treatment period and after withdrawal of treatments. In a mouse model of genetic ablation of hippocampal neurogenesis (GFAP-Tk model), neurogenesis dependent/independent effects of chronic S 47445 treatment were tested, as well as BDNF hippocampal expression. S 47445 reversed CORT-induced depressive-like state by increasing grooming duration and reversing coat state’s deterioration. S 47445 also decreased the immobility duration in TST and FST. The highest doses (3 and 10 mg/kg) seem the most effective for antidepressant-like activity in CORT mice. Furthermore, S 47445 significantly reversed the anxiety phenotype observed in OF (at 1 mg/kg) and EPM (from 1 mg/kg). In the CMS rat model, S 47445 (from 1 mg/kg) demonstrated a rapid onset of effect on anhedonia compared to venlafaxine and imipramine. In the CORT model, S 47445 demonstrated significant neurogenic effects on proliferation, survival and maturation of hippocampal newborn neurons at doses inducing an antidepressant-like effect. It also corrected CORT-induced deficits of growth and arborization of dendrites. Finally, the antidepressant/anxiolytic-like activities of S 47445 required adult hippocampal neurogenesis in the novelty suppressed feeding test contrary to OF, EPM and ST. The observed increase in hippocampal BDNF levels could be one of the mechanisms of S 47445 responsible for the adult hippocampal neurogenesis increase. Altogether, S 47445 displays robust antidepressant-anxiolytic-like properties after chronic administration through neurogenesis dependent/independent mechanisms and neuroplastic activities. The AMPA-PAM S 47445 could have promising therapeutic potential for the treatment of major depressive disorders or generalized anxiety disorders.

## Introduction

Several lines of evidence suggest glutamatergic dysfunctions in the pathophysiology of major depressive disorders (MDD). Depending on the brain region studied, glutamate levels have been found increased in the prefrontal (Hashimoto et al., 2007) and in the occipital cortices (Sanacora et al., 2004; Bhagwagar et al., 2007) or decreased in anterior cingulate cortex of MDD patients (Luykx et al., 2012). Alterations in mRNA or protein levels of glutamate receptor were measured in post-mortem studies, suggesting compromised glutamate-mediated synaptic neurotransmission in various brain areas from depressed individuals (Beneyto et al., 2007; Ding et al., 2015). Overall, these clinical reports highlight the glutamatergic synapse as well as the AMPA receptor’s activation as potential targets for therapeutic intervention (Niciu et al., 2014). Studies in animal models have confirmed these glutamatergic impairments in the context of anxiety-depression. Glutamatergic as well as gabaergic reduced activity has been observed in social defeated-mice (Veeraiah et al., 2014). Chronic unpredictable stress has been shown to impair long term potentiation in the thalamus–prefrontal cortex pathway (Quan et al., 2011) and in the hippocampus–prefrontal cortex connection (Cerqueira et al., 2007). Chronic corticosterone exposure induces a decrease in glutamatergic transmission by decreasing NR2B and GluR2/R3 expression in the ventromedian prefrontal cortex (Gourley et al., 2009).

The interest in glutamatergic dysfunction in depression has mostly grown since the observation that ketamine, a NMDA NR2B antagonist, produces at sub-anesthetic doses rapid and potent antidepressant effects in patients with treatment-resistant depression (Zarate et al., 2006; Murrough et al., 2013; Iadarola et al., 2015), and in animal models of the disease (Autry et al., 2011; Li et al., 2011). However, ketamine effects may also involve, at least in part, AMPA receptors (AMPAR) activation since its effect is blocked by an AMPA antagonist (NBQX) (Koike et al., 2011; Yamanaka et al., 2014). Additionally, recent studies suggest that antidepressant-like effects of ketamine may be mediated through its metabolite, (2R,6R)-hydroxynorketamine, that would act in a sustainable manner on AMPA receptors (Zanos et al., 2016).

Positive allosteric modulators bind to allosteric sites on AMPA receptors, slow desensitization and enhance synaptic currents through receptors, thereby promoting synaptic transmission and plasticity (Black, 2005; Lynch and Gall, 2006; Morrow et al., 2006; Pirotte et al., 2013). These modulators facilitate episodic and spatial working memories in a number of behavioral studies mostly in rodents and in monkeys studies (O’Neill et al., 2004; Black, 2005; Lynch, 2006; O’Neill and Dix, 2007; Ward et al., 2010). They have been shown to induce solely antidepressant-like responses in rodents (Li et al., 2001; Farley et al., 2010; Andreasen et al., 2015) and also synergistically with various classes of antidepressant treatments (Li et al., 2003; Andreasen et al., 2013).

Current antidepressant treatments (such as selective serotonin and or norepinephrine reuptake inhibitors) modulate AMPA- and NMDA-mediated synaptic plasticity in neuronal circuits but indirectly, which may be one of the reasons explaining the observed delay between drug administration and antidepressant efficacy.

Interestingly, similarly to antidepressant treatments, AMPA (Bai et al., 2003) and AMPA-positive allosteric modulators (AMPA-PAM) increase the levels of neurotrophins and notably BDNF *in vitro* (Lauterborn et al., 2000, 2003; Legutko et al., 2001; Jourdi et al., 2009) and in various animals models (Mackowiak et al., 2002; Rex et al., 2006; Woolley et al., 2009; Akinfiresoye and Tizabi, 2013). In the adult hippocampus, chronic antidepressant treatments are known to stimulate neurogenesis (Malberg et al., 2000) and BDNF synthesis (Nibuya et al., 1995), while genetic ablation of Bdnf hampers the mechanism of action of chronic antidepressant treatment (Adachi et al., 2008). Yet, few studies observed the characterization of neurogenic effects of AMPA-PAM, and only looked at cell proliferation/cell survival in the dentate gyrus of the hippocampus (Bai et al., 2003; Su et al., 2009). Thus it remains unclear whether this class of compounds can stimulate the whole process of adult neurogenesis and can facilitate their differentiation into mature neurons.

S 47445 (8-cyclopropyl-3-[2-(3-fluorophenyl)ethyl]-7,8-dihydro-3H-[1,3]oxazino[6,5-g][1,2,3] benzotriazine-4, 9-dione) is a novel and selective positive allosteric modulator of the AMPA receptors, with no affinity for orthosteric binding sites at AMPA, NMDA and kainate receptors (Danober et al., 2016; Giralt et al., 2017). In *Xenopus laevis* oocytes expressing rat or human AMPA receptors, S 47445 potently and selectively enhanced AMPA-evoked inward currents (EC_50_ = 6.5 μM) in a concentration-dependent manner without affecting NMDA and kainate activity (Danober et al., 2016). Such concentration-dependent potentiation by S 47445 was also observed on glutamate-evoked currents in *X. laevis* oocytes expressing human homomeric and heteromeric GluA variants with similar EC_50_. S 47445 exhibited at a low concentration a decrease of desensitization associated to an increase of the amplitude of the response and sensitivity to glutamate in HEK-293 cells, showing that S 47445 is a potent AMPA-PAM (Danober et al., 2016).

Here, we hypothesized that chronic administration of S 47445 would reduce behavioral emotionality in a mouse model of anxio/depressive-like phenotype and anhedonia induced in rat by the chronic mild stress. Given that classical antidepressant treatments are known to increase adult hippocampal neurogenesis as well as dendrite growth, we explored the stimulating effects of chronic S 47445 on cell proliferation, cell survival and neuronal maturation and arborization. We then further assessed that ablation of neurogenesis impairs antidepressant-induced behavioral response in tests that require neurogenesis using mice in which the glial fibrillary acidic protein (GFAP)-positive progenitor cells die following treatment with ganciclovir (Schloesser et al., 2009). Finally, in these mice, S 47445 induced-increase in hippocampal BDNF synthesis was maintained despite arresting neurogenesis.

## Materials and Methods

### Animals

**FIGURE 1:**
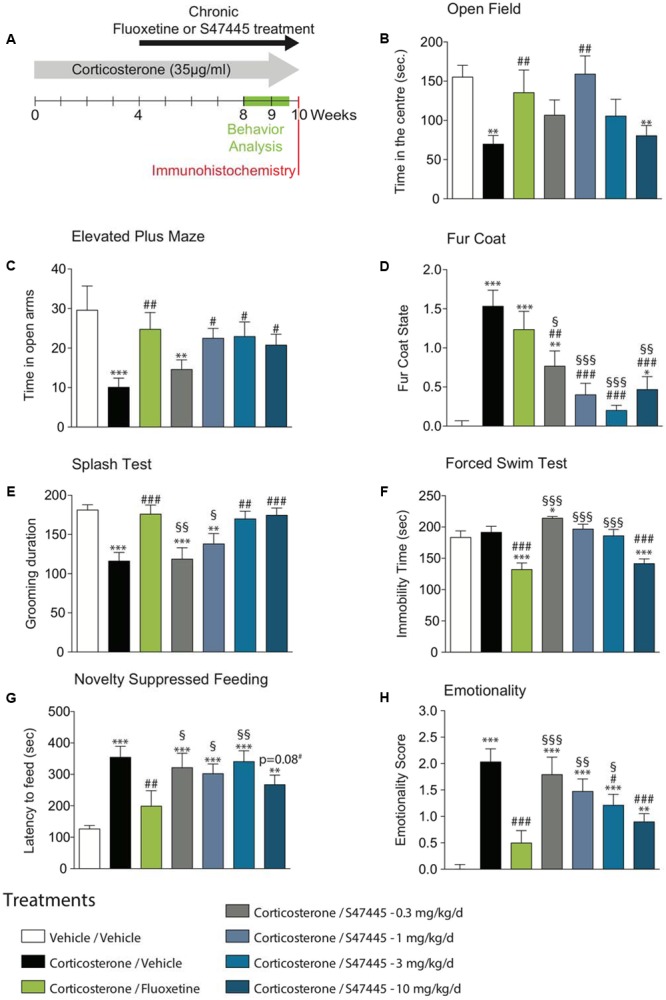
Chronic S 47445 administration has anxiolytic- and antidepressant-like effects. Timeline of experiments **(A)**. Anxiety was expressed as the time spent in the center during 30 min in the open field **(B)** and as the time spent in the open arms of the elevated plus maze **(C)**. Alteration in the fur coat state after treatments administration was measured **(D)** and confirmed in the splash test, where grooming duration over a 5 min period was measured **(E)**. Immobility time was recorded in the forced swim test **(F)**. In the novelty suppressed feeding, latency time to feed was measured **(G)**. Use of *z*-score normalization was performed to observe the effects of chronic corticosterone and/or S 47445 or fluoxetine treatment on animals behavioral emotionality **(H)**. Data are expressed as mean ± SEM (*n* = 13–15 animals/group). ^∗^*p*< 0.05; ^∗∗^*p*< 0.01 and ^∗∗∗^*p* < 0.001 for comparisons to the vehicle/vehicle-treated group. ^#^*p*< 0.05; ^##^*p*< 0.01 and ^###^*p* < 0.001 for comparisons to the corticosterone/vehicle-treated group. ^§^*p*< 0.05; ^§§^*p*< 0.01 and ^§§§^*p* < 0.001 for comparisons to the corticosterone/fluoxetine-treated group.

One hundred and five C57BL6/NTac male mice, 7–8 weeks old (25–30 g, Taconic Farms, Denmark) were used to test the behavioral and neurogenic effects of treatments in the CORT model (See timeline of experiments in **Figure 1A**). 30 transgenic GFAP-Tk+ and 30 transgenic GFAP-Tk mice aged 10–14 weeks of age were used to assess the role of neurogenesis in treatment response (Schloesser et al., 2009; **Figure 4A**). Mice were maintained under standard conditions (12/12 h light/dark cycle, lights on at 6AM, 22 ± 1°C, food and water *ad libitum*, 5 mice/cage). The protocols involving animals and their care were conducted in conformity with the institutional guidelines that are in compliance with national and international laws and policies (Council directive # 87-848, October 19, 1987, Ministère de l’Agriculture et de la Forêt, Service Vétérinaire de la Santé et de la Protection Animale, permissions # 92-256B to DJD and with the European Communities Council Directive 2010/63/UE) and in compliance with protocols approved by the Institutional Animal Care and Use Committee (CEE26 authorization 2012-099).

Male Wistar rats (*n*= 8 per group, Charles River, Germany) were brought into the laboratory 2 months before the start of the experiment. Animals were singly housed with food and water freely available, and were maintained on a 12-h light/dark cycle (lights on at 08.00) at a temperature of 22 ± 2°C. The study was conducted in compliance with the with the rules and principles of the the European Communities Council Directive 2010/63/UE and were approved by the Bioethical Committee at the Institute of Pharmacology, Polish Academy of Sciences, Krakow, Poland.

### Physiological Response to Treatment

Each week, animals were monitored for their weight and fur coat state by an observer blind to the treatment. For this latter, the score obtained characterize the alteration of the fur coat, one of the hallmark of chronic exposition to chronic CORT in C57BL6 mice (David et al., 2009). The score results from the sum of the score of five different body parts: head, neck, dorsal/ventral coat, tail and fore-/hind paws. For each body part, a score of 0 was given for good state (smooth fur), 1 for moderate degradation (fur with some spiky patches) and 2 for unkempt coat (bad fur) (Mekiri et al., 2017).

### Behavioral Analysis in Mouse

#### The Elevated Plus Maze

Behavior in the elevated plus maze was measured using a cross maze with two open and two closed arms (30 × 5 cm arms). Numbers of entries and time spent in the open arms during a 5 min test measured anxiety-related behaviors. The different parameters were recorded by a digital camera and scored by videotracking (EPM3C software, Bioseb, Vitrolles, France). The distance traveled in the maze was used as an index of locomotor activity.

#### The Open-Field Paradigm

Motor activity was quantified in Plexiglas open field boxes 43 cm × 43 cm (MED associates, Georgia, VT, United States) during a 10-min session (Guilloux et al., 2011a). Two sets of 16 pulse-modulated infrared photo beams were placed on opposite walls 2.5 cm apart to record x-y ambulatory movements. Activity chambers were computer interfaced for data sampling at 100 ms resolution. The center was defined as a 32 × 32-cm central arena. Dependent measures were: total time spent in the center, the numbers of entries into the center and distance traveled in the center divided by total distance traveled. Overall motor activity was quantified as the total distance traveled (cm).

#### The Novelty-Suppressed Feeding Test

The novelty-suppressed feeding paradigm is a conflict test that elicits competing motivations: the drive to eat and the fear of venturing into the center of a brightly lit arena. Briefly, animals were food-deprived for 24 h prior to the test. Testing was performed in a 50 cm × 40 cm × 20 cm box covered with bedding and illuminated by a 70-watt lamp. The NSF test was carried out during a 10-min period (Guilloux et al., 2013). At the time of testing, a single pellet of food (regular chow) was placed on a white paper platform positioned in the center of the box. Mice were tested individually by placing them in the corner of the maze for 10 min. The latency to eating was timed. Immediately afterward, the animal was transferred to its home cage and the amount of food consumed during the subsequent 5 min was measured, serving as a control for change in appetite as a possible confounding factor (home cage food consumption), because antidepressants are known to affect appetite.

#### Splash Test

This test consisted in squirting a 10% sucrose solution on the mouse’s snout. The grooming duration was then recorded over a 5 min period in the home cage of the animal (Ducottet and Belzung, 2004).

#### The Mouse Forced Swim Test

The forced swim test procedure was modified to enhance the sensitivity to detect the putative antidepressant-like activity of drugs (Porsolt et al., 1977; Rainer et al., 2012). Briefly, mice were placed into clear plastic buckets, 20 cm in diameter and 23 cm deep, filled up to two-thirds with water at ≈24°C. Automated scoring was done using the automated X’PERT FST (Bioseb, Vitrolles, France). Dependent variables were mobility, swimming and climbing duration.

#### The Tail Suspension Test

The tail suspension test is an antidepressant activity screening test (Steru et al., 1985) often used to test compounds that are expected to affect depression related behaviors. Mice are suspended by their tails with tape, in such a position that they cannot escape or hold on to nearby surfaces. During this test, typically 6 min in duration, the resulting escape oriented behaviors are quantified using an automated tail suspension test apparatus (Bioseb, Vitrolles, France). A specific strain gauge linked to a computer quantifies the time spent by the animal trying to escape.

#### Emotionality Measurement

Four behavioral tests (OF, EPM, NSF, and Splash test) were used to measure components of animal emotionality. *Z*-score methodology was used to investigate the potential of combining results within and across the different behavior tests for depressive/anxious-like behaviors and investigate the treatment effects in the CORT model. In the OF, the total time spent in center, the number of entries in center and the distance ratio, in the EPM, the time in open arms and the entries ratio, the latency to feed in NSF and grooming duration in Splash test were considered as emotionality parameters. For each emotionality parameter, the *Z*-score value for individual mouse was calculated the formula below:

$$\begin{array}{c} Z=\frac{X-\mu }{\sigma } \end{array}$$

which indicated how many standard deviation (σ) an observation (X) is above or below the mean of a control group. The emotionality-related data was normalized as previously described (Guilloux et al., 2011b), the veh/veh group was defined as the control. In each test, individual *Z*-score value was calculated, respectively, for each group using the means (μ) and standard deviation (σ) of the control group. The increased emotionality was defined as decreased normalized open field center activity, decreased activity in the open arms in the elevated plus maze, increased NSF latency and decreased grooming in the Splash test compared to control group means. The finally individual emotionality score across the tests and finally group emotionality score means were calculated as previously described (Guilloux et al., 2011b).

### Sucrose Consumption in the Chronic Mild Stress (CMS) Model in Rat

After a period of 3 weeks of adaptation to laboratory and housing conditions, the animals were trained to consume a 1% sucrose solution; training consisted of ten 1 h baseline tests in which sucrose was presented, in the home cage, following 14 h food and water deprivation. The sucrose intake was measured by weighing pre-weighed bottles containing the sucrose solution, at the end of the test. Subsequently, sucrose consumption was monitored, under similar conditions, at weekly intervals throughout the whole experiment. On the basis of their sucrose intakes in the final baseline test, the animals were divided into two matched groups. One group of animals was subjected to the chronic mild stress procedure for a period of eight consecutive weeks. Each week of stress regime consisted of: two periods of food or water deprivation, two periods of 45 degree cage tilt, two periods of intermittent illumination (lights on and off every 2 h), two periods of soiled cage (250 ml water in sawdust bedding), one period of paired housing, two periods of low intensity stroboscopic illumination (150 flashes/min), and three periods of no stress. All stressors were 10–14 h of duration and were applied individually and continuously, day and night. Control animals were housed in separate rooms and had no contact with the stressed animals. They were deprived of food and water for 14 h preceding each sucrose test, but otherwise food and water were freely available in the home cage. After 5 weeks, all treatments were terminated and one additional sucrose test was carried out following 1 week of withdrawal. Stress was continued throughout the period of treatment and withdrawal.

### Drugs and Treatment

#### Study in the CORT Model

Corticosterone (35 μg/ml, equivalent to about 5 mg/kg/day) or vehicle (0.45 % β-cyclodextrin, β-CD) were available *ad libitum* in the drinking water in opaque bottles to protect it from light. Corticosterone-treated water was changed every 3 days to prevent any possible degradation. S 47445 (0.3, 1, 3 and 10 mg/kg/d in 1% hydroxyethyl cellulose and 1% Tween 80 in distilled water, oral gavage, p.o.) and fluoxetine (18 mg/kg/d in distilled water, oral gavage, p.o.) were administered chronically to mice during 28 days and their effects observed in various behavioral tests. The mice were tested 24 h after the last administration.

#### Study in the CMS Rat Model

Animals received once daily administration of two vehicles (1% (w/v) hydroxyethylcellulose and 1% (v/v) polysorbate Tween 80 in distilled water and 0.9% sodium chloride aqueous solution), S 47445 (0.3, 1, 3, and 10 mg/kg/day in 1% hydroxyethyl cellulose and 1% Tween 80 in distilled water, p.o.), venlafaxine and imipramine (both at 10 mg/kg/day, i.p, in 0.9% sodium chloride aqueous solution) for 5 weeks. The volume of all administration was 1 ml/kg. The drugs were administered at 10.00 a.m. and the weekly sucrose tests were carried out 24 h following the last drug injections.

#### Study in GFAP-Tk^+^ Mice

To arrest neurogenesis, ValGanciclovir (VGCV, Roche, Indianapolis, IN, United States) – the L-valyl ester of ganciclovir - were given to GFAP-Tk^+^ positive mice from Monday to Friday during 12 weeks through the animals’ chow at a concentration of 15 mg/kg/day. The control group (*n* = 20) received regular chow. S 47445 (10 mg/kg/d in 1% hydroxyethyl cellulose and 1% Tween in distilled water, oral gavage, p.o.) and fluoxetine (18 mg/kg/d in distilled water, oral gavage, p.o.) were administered chronically to mice during 28 days and their effects observed in various behavioral tests. The mice were tested 24 h after the last administration.

All experimenters were blind of the treatment administered to animals.

### Immunohistochemistry

#### Ki67 Labeling

##### Proliferation study

For Ki67 immunostaining, sections were incubated in 0.3% triton in PBS and 10% Normal Donkey Serum (NDS). Then sections were incubated overnight at 4°C with anti-rabbit Ki67 (from Vector; 1:100). After washing with PBS, sections were incubated for 2 h with secondary antibody (1:200 biotinylated donkey anti-rabbit). Cells were counted using a BX51 microscope (Olympus, Germany).

#### 5-Bromo-2-Deoxyuridine (BrdU) Labeling

##### Survival study

Mice were administered BrdU (150 mg/kg, i.p. b.i.d. for 3 days) before the start of the different treatments and processed as described by Xia et al. (2012). BrdU^+^ cells were counted under the microscope.

#### Doublecortin (DCX) Labeling for Maturation Index Study

The immunohistochemistry protocol was adapted from David et al. (2009). DCX-positive (DCX^+^) cells were subcategorized according to their dendritic morphology: DCX^+^ cells with no tertiary dendritic processes and DCX^+^ cells with tertiary (or higher order) dendrites. The maturation index was defined as the ratio of DCX^+^ cells possessing tertiary dendrites over the total number of DCX^+^ cells.

### Sholl Analysis

The Sholl technique is used to describe neuronal arbors of isolated neurons. Sholl analysis is performed by creating a series of concentric shells around the focus of a neuronal arbor, and counts how many times connected voxels defining the arbor intersect the sampling shells. For Sholl analysis, DCX^+^ cells with tertiary, relatively untruncated dendritic branches were traced for each 35 μm hippocampal slice using Neurolucida software (MicroBrightField, Williston, VT, United States) on an Olympus BX51 microscope equipped with a motorized stage device and ×100 immersion oil objective. DCX immunohistochemistry was done to maximize the labeling of dendrites. Sholl analysis for dendritic complexity was performed using the accompanying software (NeuroExplorer; MicroBrightField, version 10), calculating dendritic complexity including dendritic length and number of intersections (branch points). Within each concentric shell from the soma, sum of length of dendrites and number of intersections characterize the overall length of the arbor and its complexity.

### Western Blot Protocol

For Western Blot analysis, mice were killed by cervical dislocation, cortex and hippocampi were rapidly dissected. The proteins were extracted in 20 mM Tris (pH 7.4), 137 mM NaCl, 2 mM EDTA (pH 7.4), 1% Triton, 25 mM β-glycerophosphate, 1 mM Na_3_VO_4_, 2 mM sodium pyrophosphate, 10% glycerol, 1 mM PMSF, 10 μg/ml aprotinin, and 10 μg/ml leupeptin]. The homogenates were centrifuged at 15,000 rpm for 20 min at 4°C.

Equal amounts of denatured proteins were loaded onto 10% or 12.5% SDS-PAGE gel and transferred on a PVDF membrane (Amersham Biosciences, Les Ulis, France). Membranes were incubated with antibodies raised against BDNF at 1:1000 (Santa Cruz, Heidelberg, Germany). β-Actin at 1: 10 000 (Santa Cruz Biotechnology, Heidelberg, Germany) was used as a loading control. Immunoreactive bands were detected using appropriate peroxide-conjugated secondary antibodies and a chemiluminescent reagent kit (Pierce Biotechnology) using a ChemiDoc XRS+ System (Bio-rad, Marnes-La-Coquette, France). Bands were quantified using Image Lab software. The densitometry values were normalized against the β-actin values as described previously (Mendez-David et al., 2013). Control conditions were taken as 100% and experimental variables were normalized with respect to this value.

### Statistical Analysis

For all experiments performed (except in the NSF), a one-way or two-way ANOVA was performed and results were expressed as mean ± SEM values. When main effects were significant, treatment comparisons were analyzed using PLSD *post hoc* test. In the NSF test, a Kaplan–Meier survival analysis was applied due to the non-normal distribution of data, as described (Samuels and Hen, 2011). Animals that did not eat during the 10-min test period were statistically censored. The Mantel-Cox log-rank test was used to evaluate differences between experimental groups.

Differences were considered significant when *P* ≤ 0.05. All analyses were conducted using Statview 5.0, Graphpad Prism or Statistica.

## Results

The complete description of statistics can be found in the Supplementals (Supplementary Table S1: studies performed in mice, Supplementary Table S2: studies performed in rats).

### Chronic S 47445 Administration has Anxiolytic-Like Effects in the CORT Mice Model

The effect of a 4-week treatment with S 47445 (0.3 to 10 mg/kg) or fluoxetine (18 mg/kg) were assessed in the Open Field (OF) and in the Elevated Plus Maze (EPM) paradigms in the CORT model (**Figure 1A**).

A one-way ANOVA on the time spent in the center, on the entries in the center and on the total ambulatory distance revealed a significant effect of treatment factor (*p* < 0.01 for all parameters tested, **Figure 1B** and **Supplementary Figures S1A,B**). Chronic exogenous corticosterone had a significant marked effect on all anxiety parameters, resulting in a significant decrease of time spent in the center (*p* < 0.01, **Figure 1B**), of number of entries in the center (*p* < 0.01, **Supplementary Figure S1A**) and of the ambulatory distance (*p* < 0.05, **Supplementary Figure S1B**). Corticosterone significantly reduced the ambulatory distance ratio (center/total, *p* < 0.01, **Supplementary Figure S1C**), hence confirming that chronic corticosterone treatment more likely affect anxiety parameters than locomotor activity. The altered behavior observed in the OF paradigm was significantly reversed by chronic S 47445 treatment at 1 mg/kg for all parameters tested, i.e., increasing significantly the time spent in the center (*p* < 0.01, **Figure 1B**), the number of entries in the center (*p* < 0.05, **Supplementary Figure S1A**), the total ambulatory distance (*p* < 0.05, **Supplementary Figure S1B**) and the ratio ambulatory distance in the center/total (*p* < 0.01, **Supplementary Figure S1C**). Chronic fluoxetine also induced an anxiolytic-like activity in corticosterone-treated animals increasing significantly the time spent in the center (*p* < 0.01, **Figure 1B**), the total ambulatory distance (*p* < 0.0001, **Supplementary Figure S1B**) and the ratio ambulatory distance in the center/total (*p* < 0.05, **Supplementary Figure S1C**).

In the elevated plus maze, a one-way ANOVA on the time spent and the number of entries in the opened arms revealed a significant effect of treatment factor (*p* < 0.01 for both parameters, **Figure 1C** and **Supplementary Figure S1D**), but not for total distance (*p* = 0.29, **Supplementary Figure S1E**) or distance ratio (*p* = 0.06, **Supplementary Figure S1F**). Chronic exogenous corticosterone in the EPM had an effect on all anxiety parameters, resulting in a decrease of the time spent (*p* < 0.001, **Figure 1C**) and in the number of entries in opened arms (*p* < 0.01, **Supplementary Figure S1D**), without affecting locomotor activity (**Supplementary Figure S1E**). Interestingly, this anxiety phenotype in the elevated plus maze paradigm was significantly reversed by chronic S 47445 at 1, 3, and 10 mg/kg as well as fluoxetine treatment for all tested parameters (**Figure 1C** and **Supplementary Figure S1D**). S 47445 and fluoxetine significantly reversed the CORT-induced decrease in time spent (*p* < 0.05 for all doses of S 47445 and *p* < 0.001 for fluoxetine) and on the number of entries in opened arms (*p* < 0.05 at 1 mg/kg; *p* < 0.01 at 3 mg/kg and *p* < 0.05 at 10 mg/kg for S 47445 and *p* < 0.001 for fluoxetine).

### Chronic S 47445 Administration has Antidepressant-Like Effects in the CORT Mice Model

The coat state of the animals, a well-validated index of a depressed-like state was next assessed (Santarelli et al., 2003). Long-term glucocorticoid exposure, similar to chronic stress induced physical changes including deterioration of the coat state. A one-way ANOVA on coat state evaluation at week 8 revealed a significant effect of treatment (*p* < 0.0001, **Figure 1D**). Chronic corticosterone strongly altered the fur coat of mice (*p* < 0.0001), an effect that was prevented by chronic S 47445 at all doses tested in a dose dependent manner (0.3 mg/kg *p* < 0.01, 1 mg/kg *p* < 0.0001, 3 mg/kg *p* < 0.0001 and 10 mg/kg *p* < 0.0001) whereas fluoxetine had no effect.

The association between fur coat alteration and grooming behavior was investigated using the splash test (**Figure 1E**). A one-way ANOVA revealed a significant effect of treatment factor on the grooming duration (*p* < 0.0001). Chronic CORT decreased grooming duration (*p* < 0.0001), an effect that was reversed after a 4-week fluoxetine treatment (*p* < 0.001), confirming previous reports (David et al., 2009; Rainer et al., 2012). At 3 and 10 mg/kg, a chronic treatment with S 47445 increased grooming duration and also reversed such depressive-like state induced by corticosterone (*p* < 0.01 and *p* < 0.001, respectively).

Animals were then tested, respectively, in two paradigms predictive of an antidepressant-like activity, the mouse Forced Swim test (FST) and the tail suspension test (TST). In the FST, a one-way ANOVA on the immobility duration revealed a significant effect of treatment (*p* < 0.0001, **Figure 1F**). In the TST, a one-way ANOVA on the immobility duration in the TST revealed a significant effect of treatment (*p* < 0.05, **Supplementary Figure S1G**). As previously reported (David et al., 2009), chronic corticosterone had no effect on immobility duration either in both tests. Fluoxetine and S 47445 at 10 mg/kg decreased the immobility duration indicating an antidepressant-like activity in the FST (*p* < 0.0001 for the two compounds, **Figure 1F**). This antidepressant-like activity was confirmed in the TST for fluoxetine and S 47445 at 3 mg/kg (*p* < 0.05).

### Chronic S 47445 Administration Reverses Anxio-Depressive Phenotype in a Neurogenesis-Dependent Task in the CORT Model

The novelty-suppressed feeding is a behavioral task that reveals the neurogenesis-dependent effects of antidepressants. In this test, it was explored whether S 47445, as fluoxetine, was able to reverse the anxiety/depressive-like state induced by corticosterone. A one-way ANOVA on the latency to feed revealed a significant effect of treatment (*p* < 0.0001, **Figure 1G**). Chronic corticosterone increased the latency to feed (*p* < 0.0001). A survival curve analysis (**Supplementary Figure S1H**) confirmed that chronic corticosterone induced an anxio/depressive-like phenotype. S 47445 at the highest dose induced a trend for a decrease in the latency to feed (*p* = 0.08) compared to corticosterone-treated mice. Such effect of S 47445 on latency to feed was further confirmed with a significant effect at 10 mg/kg of S 47445 in the experiment using GFAP-TK^-^ mice (See *infra*). Fluoxetine, as previously reported decreased the corticosterone-induced increase in latency to feed (*p* < 0.01). The food consumption was also followed in the home cage over a period of 5-min. No correlation between the latency to eat the pellet during the test *vs*. in the home cage was observed.

### Chronic S 47445 Dose-Dependently Decreases the Corticosterone-Induced Increase in Emotionality

A one-way ANOVA on emotionality scores revealed a significant effect of treatment factor (*p* < 0.0001, **Figure 1H**). As previously described (Guilloux et al., 2011b), emotionality *Z*-score normalization across behavioral tests showed a strong elevated emotionality induced by corticosterone treatment in mice compared to normal group Veh/Veh (*p* < 0.001, **Figure 1H**). This high emotionality was significantly reduced by chronic treatment with either fluoxetine (*p* < 0.001) or S 47445 in a dose dependent manner (0.3 mg/kg: *p* = 0.46; 1 mg/kg: *p* = 0.08; 3 mg/kg: *p* < 0.05; 10 mg/kg: *p* < 0.001).

### S 47445 has a Rapid Onset of Effect on Anhedonia in the Rat CMS Model

**FIGURE 2:**
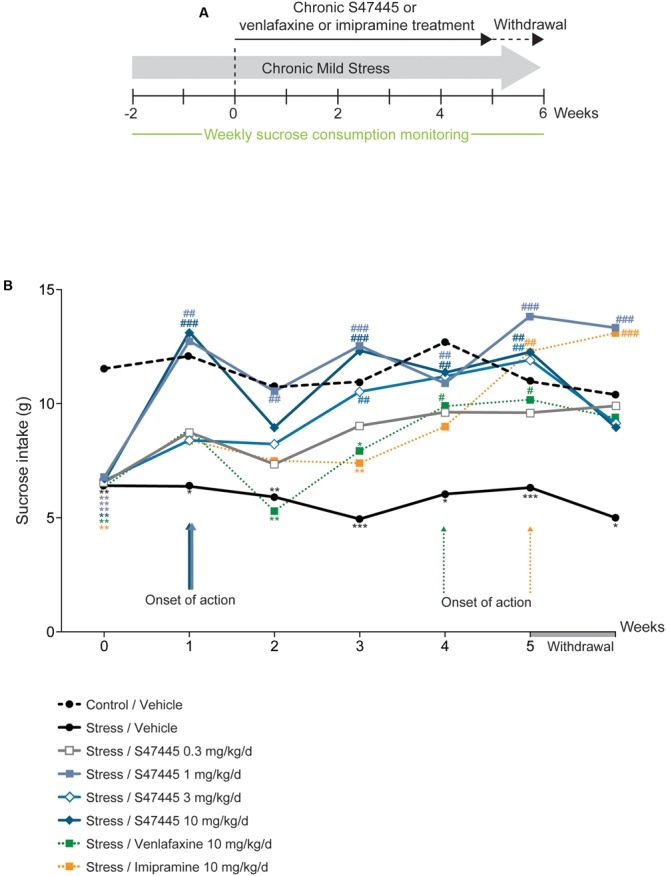
Chronic S 47445 restores stress-induced anhedonia in the CMS model. Timeline of experiments **(A)**. The treatment effects following administration with S 47445 (0.3 to 10 mg/kg) on sucrose intake in CMS-induced anhedonia were compared to those of venlafaxine or imipramine (10 mg/kg) **(B)**. Data are expressed as mean (*n* = 8 animals/group). ^∗^*p*< 0.05; ^∗∗^*p*< 0.01 and ^∗∗∗^*p* < 0.001 for comparisons to the control/vehicle-treated group. ^#^*p*< 0.05; ^##^*p*< 0.01 and ^###^*p* < 0.001 for comparisons to the CMS/vehicle-treated group.

In order to further assess the antidepressant-like properties of S 47445, the efficacy of the compound was investigated in the chronic mild stress rat model (**Figure 2A**). Sucrose consumption for rats treated with the vehicle corresponding to venlafaxine or imipramine treatments did not differ significantly from rats treated with the vehicle corresponding to S 47445, either in basal or after chronic mild stress (only results for vehicle corresponding to S 47445 are shown, **Figure 2B** and Supplementary Table S2). A two-way ANOVA analysis revealed a significant effect of treatment (*p* < 0.0001), a significant effect of time (*p* < 0.0001) but no interaction between these factors (*p* = 0.063, **Figure 2B** and Supplementary Table S2). Chronic mild stress in rats induced a basal decrease in sucrose consumption that was maintained from week 0 to 5 (*p* < 0.0001 for each timepoint, **Figure 2B**). Chronic S 47445 treatment antagonized the decrease in sucrose intake induced by stress, with significant effect starting from 1 week of treatment (S 47445 1 or 10 mg/kg: *p* < 0.001, **Figure 2A**). After 5 weeks of treatment, the doses tested 1 to 10 mg/kg reverted the stress-induced decrease in sucrose intake. After treatment withdrawal, the effect was maintained at similar level with the dose of 1 mg/kg of S 47445 (**Figure 2B**). Comparatively, imipramine and venlafaxine reversed the decrease of sucrose intake only after 4 weeks of treatment (*p* < 0.05 for both treatment, **Figure 2B**).

### Chronic S 47445 Administration Stimulates Cell Proliferation and Survival in the Dentate Gyrus of the Hippocampus in the CORT Model

Adult hippocampal neurogenesis has been shown to participate in the anxiolytic/antidepressant-like activity of antidepressant treatments. Thus, it was examined whether a chronic treatment with S 47445 could induce neurogenic effects.

**FIGURE 3:**
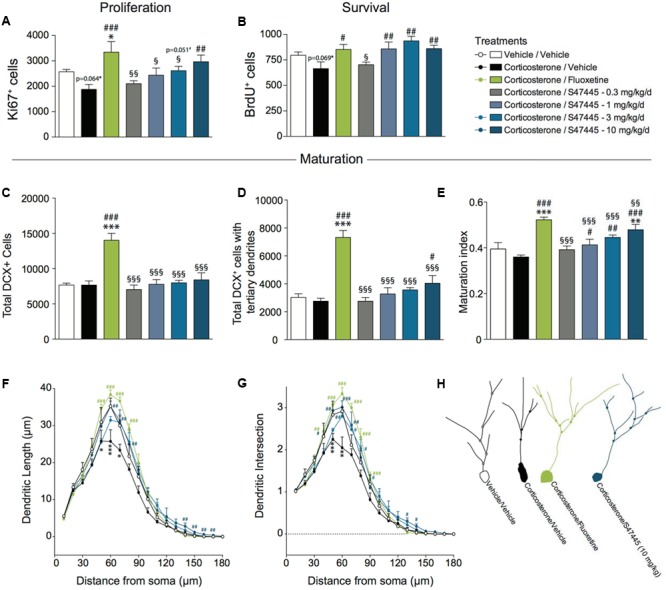
Chronic S 47445 administration stimulates neurogenesis in the dentate gyrus of the hippocampus. The effects of 28 days of treatment with S 47445 (0.3 to 10 mg/kg) on cell proliferation **(A)**, cell survival **(B)**, and newborn cell maturation **(C–E)** were compared to those of vehicle and fluoxetine (18 mg/kg) in corticosterone-treated animals. Maturation was characterized by the total number of DCX^+^ cells **(C)**, the number of DCX^+^ cells with tertiary dendrites **(D)** and the maturation index of newborn granule cells **(E)** (*n* = 4–6 animals per group). The effects of tested treatments on dendritic length **(F)** and the number of intersection **(G)** were measured (*n* = 3 mice/group, 20 cells/mouse) using a Sholl analysis of DCX^+^ neurons **(H)**. ^∗^*p*< 0.05; ^∗∗^*p*< 0.01 and ^∗∗∗^*p* < 0.001 for comparisons to the vehicle/vehicle-treated group. ^#^*p*< 0.05; ^##^*p*< 0.01 and ^###^*p* < 0.001 for comparisons to the corticosterone/vehicle-treated group. ^§^*p*< 0.05; ^§§^*p*< 0.01 and ^§§§^*p* < 0.001 for comparisons to the corticosterone/fluoxetine-treated group.

A one-way ANOVA on the proliferation of newborn cells in the whole hippocampus revealed a significant effect of treatment (*p* < 0.01, **Figure 3A** and **Supplementary Figure S2A**). Corticosterone treatment tended to decrease proliferation (-27%, *p* = 0.064). A 4-week fluoxetine treatment increased cell proliferation (+78%, *p* < 0.001) confirming previous report (David et al., 2009). Interestingly, S 47445 treatment reversed significantly the decrease in newborn cells induced by corticosterone treatment at 10 mg/kg (+58%, *p* < 0.01). S 47445 at 3 mg/kg tended to have similar effects (+ 39%, *p* = 0.051).

A one-way ANOVA on the survival of newborn cells in the whole hippocampus revealed a significant effect of treatment (*p* < 0.01, **Figure 3B** and **Supplementary Figure S2B**). Chronic corticosterone treatment tended to decrease survival (*p* = 0.069). Chronic treatment with either fluoxetine or S 47445 at 1, 3, 10 mg/kg increased the survival of newborn cells in the dentate gyrus of adult hippocampus (fluoxetine: +28%, *p* < 0.05; S 47445: 1 mg/kg: +29%, *p* < 0.01; 3 mg/kg: +40%, *p* < 0.001; 10 mg/kg: +29%, *p* < 0.01).

### Chronic S 47445 Administration Stimulates Neuronal Maturation in the Dentate Gyrus of the Hippocampus in the CORT Model

Furthermore, the effects of S 47445 or fluoxetine were assessed on dendritic maturation in the CORT model. A one-way ANOVA on the total number of DCX^+^ cells, on the total number of DCX^+^ cells with tertiary dendrites or the maturation index revealed a significant effect of treatment (*p* < 0.0001 for all analysis performed, **Figures 3C–E**). Chronic treatment with corticosterone had no effects on any of these parameters. A chronic treatment with fluoxetine increased the total number of DCX^+^ cells (*p* < 0.0001, **Figure 3C**), DCX^+^ cells with tertiary dendrites (*p* < 0.0001, **Figure 3D**) and the maturation index (*p* < 0.0001, **Figure 3E**). While S 47445 did not change the total number of DCX^+^ cells, it significantly increased the number of DCX^+^ cells with tertiary dendrites at 10 mg/kg (*p* < 0.05, **Figure 3D**). Moreover, S 47445 increased the maturation index for newborn neurons in the dentate gyrus of the hippocampus (+14%, *p* < 0.05; +23%, *p* < 0.01 and +32%, *p* < 0.001 for 1, 3, and 10 mg/kg, respectively; **Figure 3E**).

A Sholl analysis was then performed to assess the dendritic complexity and arborization of DCX^+^ cells (**Figures 3F–H**). A one-way ANOVA on the dendritic length revealed a significant effect of the treatment at 10 μm (*p* < 0.01), and from 40 to 90 μm (*p* < 0.05 or less, except at 40 μm *p* = 0.056 and at 90 μm *p* = 0.0596), and from 120 to 170 μm (*p* < 0.05 or less) (**Figure 3F** and **Supplementary Figure S2C**). Moreover, a one-way ANOVA on the dendritic intersection revealed a significant effect of the treatment from 40 to 80 μm (*p* < 0.05 or less), from 120 to 140 μm (*p* < 0.05 or less) and 160 μm (*p* < 0.05) (**Figure 3G** and **Supplementary Figure S2D**).

*Post hoc* analyses revealed that a chronic corticosterone treatment significantly decreased dendritic length from 50 to 70 μm of the soma as well as number of intersection from 40 to 60 μm of the soma. In contrast, 4 weeks of S 47445 treatment at 3 and 10 mg/kg in corticosterone-treated animals significantly enhanced both dendritic length (from 60 to 80 μm and at 140–150 μm) and the number of intersections (from 40 to 80 μm and at 130–140 μm) similarly to fluoxetine (from 50 to 90 μm for length and from 40 to 80 μm for intersection).

### S 47445 Anxiolytic/Antidepressant-Like Effects Are Mediated by Neurogenesis-Dependent and Independent Effects

**FIGURE 4:**
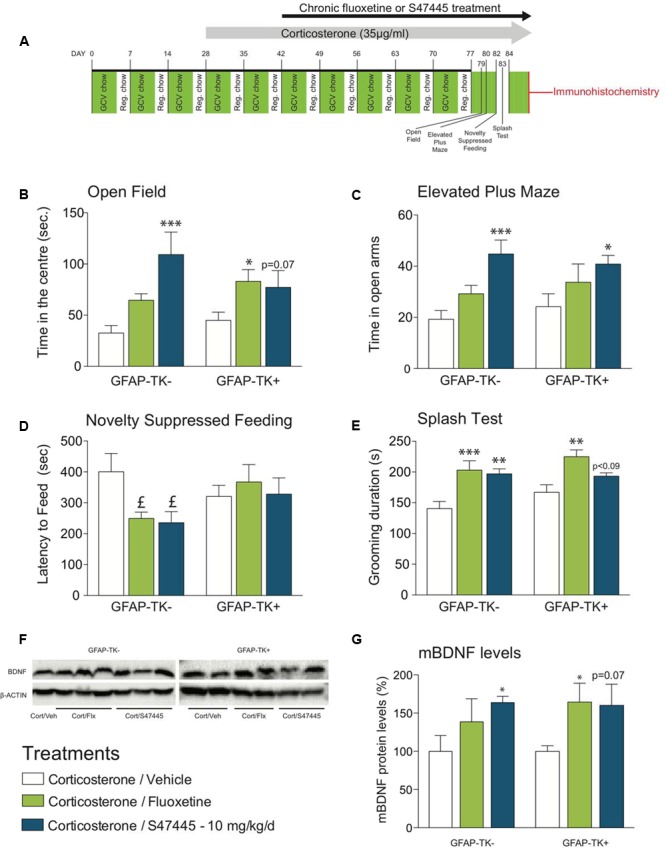
Chronic S 47445 administration has anxiolytic/antidepressant-like effects in an animal model of disrupted hippocampal neurogenesis. Timeline of experiments **(A)**. Anxiety was expressed as the time spent in the center **(B)** during 30 min in the open field and as time spent in the open arms in the elevated plus maze **(C)**. In the novelty suppressed feeding, latency time to feed was measured **(D)**. Chronic fluoxetine or S 47445 treatment effects on grooming behavior were explored in the splash test **(E)** Data are expressed as mean ± SEM (*n* = 13–15 animals/group). Representative images of whole mature BDNF detection using western blotting analyses **(F)**. mBDNF-levels were measured in the hippocampus of GFAP-TK- and GFAP-TK+ mice **(G)**. Data are expressed as mean ± SEM (*n* = 4–5 animals/group). ^∗^*p*< 0.05; ^∗∗^*p*< 0.01 and ^∗∗∗^*p* < 0.001 for comparisons to the corticosterone/vehicle-treated group within genotype. pound; *p*< 0.05 for planned comparisons to the corticosterone/vehicle-treated group within strain using Student *T*-tests analyses.

The effect of a 4-week treatment with S 47445 (10 mg/kg) or fluoxetine (18 mg/kg) were assessed in the OF, EPM, NSF, Splash Test paradigms in corticosterone-treated GFAP-TK^-^ and GFAP-TK^+^ mice (**Figure 4A**). In GFAP-TK^+^ mice, neurogenesis was ablated as the glial fibrillary acidic protein (GFAP)-positive progenitor cells die following treatment with ganciclovir (Schloesser et al., 2009). This aspect was confirmed by the large decrease (-71%) of proliferation of newborn cells in the whole hippocampus (two-way ANOVA on genotype factor: *p* < 0.001, no effect of treatment factor: *p* = 0.077, **Supplementary Figures S3A,B**).

Regarding OF, a two-way ANOVA on time in the center revealed no genotypes’ factor effect (*p* = 0.11) but a treatment factor effect (*p* < 0.001, **Figure 4B**). In GFAP-TK^-^ mice, chronic S 47445 increased the time spent in the center (*post hoc analysis p* < 0.0001, **Figure 4B**) while for fluoxetine only a trend was observed (*post hoc analysis p* = 0.088). In GFAP-TK^+^ mice, treatment effects were also comparable as chronic S 47445 tended to increase the time spent in the center (*post hoc analysis p* = 0.07, **Figure 4B**) while fluoxetine effects were significant (*post hoc analysis p* < 0.05). Regarding the number of entries in the center, a two-way ANOVA revealed an effect of genotype factor (*p* = 0.0065) but no treatment factor effect (*p* = 0.056) nor interaction (*p* = 0.59, **Supplementary Figure S4A**). Both genotype factor (*p* = 0.0014) and treatment factor (*p* = 0.0097) had an effect on ambulatory activity in the open-field, mainly supported by the hyperactivity observed in the GFAP-TK^+^ mice treated with fluoxetine (*post hoc analysis p* < 0.01, **Supplementary Figure S4B**). A treatment factor effect (but not of genotype) was observed on the ambulatory distance ratio (treatment: *p* = 0.0001, genotype: *p* = 0.25, interaction: *p* = 0.75). Chronic fluoxetine and S 47445 significantly increased the ambulatory distance ratio (center/total, *post hoc analysis p* < 0.01, **Supplementary Figure S4C**), hence confirming that S 47445 treatment more likely affects anxiety parameters than locomotor activity. In the elevated plus maze, the two-way ANOVA revealed no genotype’ effect for time in open arms (*p* = 0.64), open arm entries (*p* = 0.85) and entries ratio (*p* = 0.12) but a treatment factor effect for these parameters (*p* = 0.0003, *p* < 0.05 and *p* < 0.01, respectively, Supplementary Table S1). Chronic S 47445 increased the time in open arms in both genotypes (GFAP-TK^-^: *post hoc analysis p* < 0.001; GFAP-TK^+^: *p* < 0.05, **Figure 4C**) and only a trend for the number of entries in the open-arms was observed (**Supplementary Figure S4D**). Finally, the entries ratio was significantly increased by S 47445 in GFAP-TK^+^ (*post hoc analysis p* < 0.05, **Supplementary Figure S4E**). Similar to the observations performed in the open field, chronic fluoxetine increased the locomotor activity in GFAP-TK^+^ mice (*post hoc analysis p* < 0.01, **Supplementary Figure S4F**). Collectively, OF and EPM paradigms revealed that ablating neurogenesis does not affect the anxiolytic-like effect of antidepressant treatments in these tests.

In the NSF, fluoxetine or chronic S 47445 only decreased the latency to feed in mice with unimpaired neurogenesis (GFAP-TK^-^: *p* < 0.05 for both compounds, using a planned comparison *t*-test, **Figure 4D**), without any impact on food consumption as no correlation between the latency to eat the pellet during the test versus food consumption in the home cage was observed. Here, we confirmed in GFAP-TK^+^ mice that ablating neurogenesis blocks antidepressant effect, revealing the neurogenesis-dependent effects of antidepressant treatments.

Finally, in the splash test, the two-way ANOVA revealed no genotype factor effect (*p* = 011) but a treatment effect (*p* < 0.0001). Chronic fluoxetine increased the grooming duration in both genotypes (*post hoc analysis* in GFAP-TK^-^: *p* < 0.0001 and GFAP-TK^+^: *p* < 0.01, **Figure 4E**). Chronic S 47445 induced similar effects in GFAP-TK^-^ mice (*post hoc analysis p* < 0.01), only a trend was observed in GFAP-TK^+^ mice (*post hoc analysis p* = 0.0882).

### S 47445 Increases BDNF Levels in the Whole Hippocampus

BDNF protein levels in the hippocampus have been shown to be sensitive to stress and corticosterone exposure. This neurotrophin is restored/increased after chronic exposure to antidepressants. Here, we show that ablation of neurogenesis in the GFAP-TK model had no effect on whole hippocampus BDNF levels (two way ANOVA genotype factor *p* = 0.67, **Figures 4F,G**). However, a treatment factor was observed (*p* < 0.05). S 47445 treatment (10 mg/kg) induced the synthesis of the mature form of BDNF protein in GFAP-TK^-^ mice (*post hoc analysis p* < 0.05). Interestingly, despite the ablation of neurogenesis in the GFAP-TK^+^ model, this effect of S 47445 on mature BDNF protein was nearly maintained (*post hoc analysis p* = 0.07, (**Figure 4G**). Similar BDNF increase was observed after fluoxetine treatment induced in GFAP-TK^+^ mice (*post hoc analysis p* < 0.05, **Figure 4G**).

## Discussion

The above findings demonstrate the antidepressant/anxiolytic-like activity for S 47445, a novel AMPA positive allosteric modulator, after chronic administration using two different behavioral animal models: one anxio-depressive phenotypic mice model based on the elevation of exogenous glucocorticoid by administration of corticosterone in drinking water, the second one inducing an anhedonic state in rats following chronic mild stress.

Moreover, we provide evidence that S 47445 exerts a dose-dependent effect on cell survival, cell proliferation and maturation index in the dentate gyrus of the hippocampus in the CORT mice model. This pro-neurogenic effect of S 47445 is confirmed by increasing the ratio of mature cells with tertiary dendrites. Using GFAP-TK model of genetic ablation of neurogenesis, it was observed that some antidepressant-like effects of S 47445 are either neurogenesis dependent or independent. We also provide evidence that S 47445 increased mature BDNF protein expression even in animals presenting a strong decrease of neurogenesis. Collectively, the current study showed that S 47445 presented a good antidepressant efficacy with a faster onset of action toward anhedonia in rats.

Firstly, the interest of the chronic corticosterone mice model relies on the induction of a robust alteration in emotion-related behavior of the animals (David et al., 2009; Guilloux et al., 2011b; Rainer et al., 2012; Mendez-David et al., 2014; Hill et al., 2015), mainly related to the HPA-axis dysfunction (David et al., 2009; Hill et al., 2015) and the physiological associated changes (David et al., 2009). Cognitive deficits (Darcet et al., 2014, 2016) and altered circadian/sleep rhythms are also described in this model (David et al., 2009; Rainer et al., 2012; Le Dantec et al., 2014). Collectively, all these aspects mimic in part the symptoms observed in human MDD disorders. In the present study, in order to correctly assess the antidepressant/anxiolytic-like activities of the drugs, various behavioral read outs have been performed in corticosterone mice (grooming behavior, TST, FST and splash test for assessing antidepressant-like effects; and EPM, OF for anxiolytic-like effects and NSF for both components). Interestingly, S 47445 at all doses improved the motivation toward self-care behavior assessed by the fur coat state of the animals in a dose-dependent manner while chronic fluoxetine tends to do so without reaching significance in this study (Rainer et al., 2012; David et al., 2016). This effect of S 47445 suggests a restoration of a normal grooming behavior, as further detected in the splash test. In this latter test, the viscosity of the sucrose solution as well as its palatability stimulate and motivate the self-grooming of animals (Ducottet and Belzung, 2004). This observation is also supported by S 47445 efficacy on anhedonia in the CMS model in rat. The antidepressant-like activity of S 47445 was also confirmed in the forced swim (10 mg/kg) and tail suspension tests (3 mg/kg) in the corticosterone mice. While these tests may not be relevant for monitoring “behavioral despair” (Nestler and Hyman, 2010; de Kloet and Molendijk, 2016), they are still considered as screening models for assessing antidepressant-like effect (Petit-Demouliere et al., 2005).

In addition, S 47445 demonstrated anxiolytic-like effects in the elevated plus maze (at 1 mg/kg) and open field (from 1 mg/kg) tests in the model of depression/anxiety corticosterone mice. Thus, S 47445 differs from another AMPA-PAM, LY392098, that presented antidepressant properties but did not rescue stress-induced anxiety-like behaviors (Farley et al., 2010).

Collectively, in the CORT model, chronic S 47445 reduces behavioral emotionality in a dose-dependent manner, affecting both CORT-induced depression and anxiety-like behavior (**Figure 1**) without altering locomotor activity in mice. The highest doses (3 and 10 mg/kg) seem the most effective and 10 mg/kg of S 47445 demonstrated similar efficacy than the antidepressant fluoxetine. Further, S 47445 also induced a fast onset of action on anhedonia in the CMS model (**Figure 2**), a well-established and widely used animal model of depression in which anhedonia is considered to reflect the decrease ability to experience pleasure as a core symptom of depression (Willner, 2017). Indeed, chronic S 47445 (1 and 10 mg/kg) presented a fast onset of action by reducing anhedonia after 1 week of treatment, while venlafaxine and imipramine effects appeared only after 4 weeks of administration. This faster onset of action of S 47445 was also observed with other AMPA positive allosteric modulators. Indeed, CX691 and CX731 displayed a significant antidepressant-like effect in the submissive behavior model in rats after the 1st week of treatment whereas fluoxetine exerts such effect after 2 weeks (Knapp et al., 2002). Additionally, our results showed that S 47445 did not exert any effect in non-stressed animals, suggesting that, as venlafaxine or imipramine, its antidepressant-like effects are observed in behavioral modified situation.

Lastly, the observed antidepressant-like effect of S 47445 observed in two different rodent models corroborates previous results obtained with other AMPA-positive allosteric modulators as described with LY392098, PISMD, LY451646 (Li et al., 2001; Samuels and Hen, 2011; Andreasen et al., 2013, 2015). Yet the clinical efficacy of AMPA-PAMs remains to be confirmed. To date, only one phase 2 clinical trial evaluated the efficacy of Org26576 in depressed subjects and report a lack of significant effect of the molecule compared to placebo on patients symptomatology (Nations et al., 2012).

Another relevant factor is the critical role of adult hippocampal neurogenesis in the antidepressant-like effects. Antidepressant effects have been associated with changes in adult hippocampal neurogenesis (Santarelli et al., 2003; Airan et al., 2007; Wang et al., 2008) and increasing adult hippocampal neurogenesis promotes resilience to the effects of CORT (Hill et al., 2015). We found that chronic S 47445 treatment increased both proliferation (10 mg/kg) and survival (1, 3, and 10 mg/kg) of cells in the adult dentate gyrus of the hippocampus. These results thus confirm prior observation of proneurogenic effect of other AMPA-PAMs, LY451646, and Org 26576 since these compounds have been shown to increase cell survival and proliferation in the dentate gyrus after chronic administration in rats (Bai et al., 2003; Su et al., 2009).

However, not all antidepressant effects are related to neurogenesis. Here, we show that arresting neurogenesis using the GFAP-Tk genetic model, blocked effects of both chronic fluoxetine or S 47445 administration in the novelty suppressed feeding paradigm, a test previously shown to reveal the neurogenesis-dependent effect of antidepressant (David et al., 2009; Mendez-David et al., 2014). Inversely, the splash test as well as the anxiolytics effects assessed by OF and EPM were independent of neurogenesis.

In addition, we extended here the neurogenic study of effects of AMPA-PAMs since effect on synaptogenesis is perhaps more important than neurogenesis for the early stages of recovery. We showed that chronic administration of S 47445 (at 3 and 10 mg/kg) stimulated maturation of immature granule cells in the dentate gyrus as observed using maturation index. Of interest, a Sholl analysis revealed that S 47445, as fluoxetine, increased the dendritic length and the number of intersection of DCX^+^ cells, thus suggesting a more complex dendritic arborization. Finally, the dendrites effects on branching and length induced by S 47445 confirms the observations with the Ampakines CX929 which was recently shown to reverse the dendritic retraction observed in middle aged rats (Lauterborn et al., 2016).

As classically observed with chronic antidepressant treatment, S 47445-induced effects on dendritic morphology, maturation, and behavioral response may be mediated by neurotrophic factors, and especially brain-derived neurotrophic factor (BDNF) (Saarelainen et al., 2003; Monteggia et al., 2004; Castren and Kojima, 2017). Indeed, the variations of mBDNF expression induced by chronic S 47445 administration (10 mg/kg) or fluoxetine observed herein are associated with changes in cell proliferation in GFAP-Tk^-^ mice (**Supplementary Figure S3**). However, results obtained in GFAP-Tk^+^ mice showed that ablating neurogenesis does not significantly impact the antidepressant-induced increase in mBDNF levels in the whole hippocampus. These results are in line with prior observation that irradiation did not affect exercise-induced increase in BDNF levels in the hippocampus (Fuss et al., 2010). To our knowledge, there is yet no report of effects of antidepressant effect on BDNF synthesis in animals with disrupted neurogenesis.

While the role of adult hippocampal neurogenesis in the physiopathology of mood disorders remains in debate (Miller and Hen, 2015), several studies suggest a relationship between neurogenesis and hippocampal-dependent cognitive functions. Indeed, ablation of neurogenesis in the DG has been shown to impair hippocampus-dependent learning (Shors et al., 2001; Madsen et al., 2003) and affect context discrimination representations (Treves et al., 2008; Sahay et al., 2011). While AMPA-positive allosteric modulators have been shown to improve performance in animal models of learning and memory (Black, 2005; Woolley et al., 2009), thus the beneficial effects of S 47445 on learning deficits observed in the CORT model (Darcet et al., 2014) remains to be tested. Nevertheless, S 47445 exhibited procognitive efficacy in multiple *in vivo* models by improving episodic-like memory and working memory in both adult and aged rodents (Louis et al., 2015).

Overall, these results support the interest of glutamatergic-based therapeutic strategies, and more specifically AMPAR potentiation to alleviate depressive/anxiety phenotype (Alt et al., 2006) as suggested also by several work using ketamine or its metabolite (2R,6R)-hydroxynorketamine (Maeng et al., 2008; Koike et al., 2011; Zhou et al., 2014; Zanos et al., 2016).

## Conclusion

Our study provides evidence that chronic treatment with S 47445 exerts antidepressant-like effects throughout neurogenesis dependent and independent mechanism, with a faster mechanism of action compared to traditional antidepressant. S 47445 demonstrated also in this study anxiolytic-like effects as well as neuroplastic activities. Altogether, it supports the development of AMPA-positive allosteric modulation as new mechanism for fast antidepressant action.

## Author Contributions

EM, SB, MP, and DD designed the study. IM-D, J-PG, LT, and MP conducted experiments. IM-D, J-PG, and DD performed data analysis. All authors wrote or contributed to the writing of the manuscript.

## Conflict of Interest Statement

DD serves as a consultant for Lundbeck, Roche, and Servier. AG receives research funding from Servier. MP receives research funding from Servier. EM and SB are Servier employees. The other authors declare that the research was conducted in the absence of any commercial or financial relationships that could be construed as a potential conflict of interest.

## Supplementary Material

FIGURE S1 Effects of chronic S 47445 or fluoxetine treatment on additional emotionality measurements. Behavioural effects of S 47445 were studied after 28 days of administration at doses of 0.3, 1, 3, and 10 mg/kg, compared to those of vehicle and fluoxetine (18 mg/kg) in corticosterone treated animals. Additional measures of anxiety-related behavior were the number of entries in the centre **(A)**, the ambulatory distance **(B)**, and the distance ratio in the centre of the open-field **(C)**. In the elevated plus maze, the number of entries in open arms **(D)**, the total distance **(E)**, and the entries ratio **(F)** were recorded. Immobility time in the tail suspension test was measured **(G)**. Data are expressed as mean ± SEM (*n* = 13–15 animals/group). In the novelty suppressed feeding, latency time to feed was measured and expressed as cumulative survival with percentage of animals that have not eaten over 10 min **(H)**. ^∗^*p*< 0.05; ^∗∗^*p*< 0.01 and ^∗∗∗^*p* < 0.001 for comparisons to the vehicle/vehicle-treated group. ^#^*p*< 0.05; ^##^*p*< 0.01 and ^###^*p* < 0.001 for comparisons to the corticosterone/vehicle-treated group.

FIGURE S2 Chronic S 47445 administration stimulates cell proliferation, cell survival and dendritic arborization in the dentate gyrus of the hippocampus. The effects of 28 days of treatment with S 47445 on cell proliferation, cell survival and dendritic complexity were compared to those of vehicle and fluoxetine. Representative illustrations of Ki67 **(A)** or DCX **(B)** staining following chronic administration of either fluoxetine or S 47445. The effects of tested treatments on dendritic length **(C)** and the number of intersection **(D)** were measured (*n* = 3 mice/group, 20 cells/mouse) using a Sholl analysis of DCX^+^ neurons. ^∗^*p*< 0.05; ^∗∗^ *p*< 0.01 and ^∗∗∗^ *p* < 0.001 for comparisons to the vehicle/vehicle-treated group. ^#^ *p*< 0.05; ^##^ *p*< 0.01 and ^###^ *p* < 0.001 for comparisons to the corticosterone/vehicle-treated group. ^§^*p* < 0.05; ^§§^*p* < 0.01 and ^§§§^*p* < 0.001 for comparisons to the corticosterone/fluoxetine-treated group.

FIGURE S3 Effects of chronic S 47445 administration on cell proliferation in the dentate gyrus of GFAP-TK+ and GFP-TK- mice. The effects of 28 days of treatment with S 47445 (10 mg/kg) on cell proliferation in the dentate gyrus of the hippocampus were compared to those of vehicle and fluoxetine in GFAP-TK+ and GFAP-TK- mice, using KI67 immunostaining. Cell proliferation was strongly decreased in GFAP-TK+ mice compared to GFAP-TK- mice **(A)**, while fluoxetine or S 47445 increased cell proliferation in GFAP-TK- mice only. Representative illustrations of Ki67 staining following chronic administration of either fluoxetine or S 47445 (10 mg/kg) **(B)**. Data are expressed as and mean ± SEM. (*n* = 4–5 animals/group). ^∗^*p* < 0.05; ^∗∗^*p* < 0.01 for comparisons to the corticosterone/vehicle-treated group within genotype. ^#^ *p*< 0.05 for genotype comparison within the same treatment group.

FIGURE S4 Effects of chronic S 47445 administration on physiological and additional behavior outcomes in GFAP-TK+ and GFP-TK- mice. The effects of 28 days of treatment with S 47445 (10 mg/kg) on behavioral outcomes were compared to those of vehicle and fluoxetine in GFAP-TK+ and GFAP-TK- mice. In the open field, anxiety was expressed as the number of entries in the centre **(A)**. Locomotor activity was reported as total ambulatory distance traveled **(B)** and using the distance ratio **(C)**. In the elevated plus maze, anxiety was expressed as number of entries in the open arms **(D)** and as entries ratio **(E)**. Locomotor activity was also monitored in the elevated plus maze by measuring total distance **(F)**. Data are expressed as mean ± SEM (*n* = 13–15 animals/group). ^∗^*p* < 0.05 and ^∗∗^*p*< 0.01 for comparisons to the corticosterone/vehicle-treated group within the same genotype. ^#^ *p*< 0.05; ^##^ *p*< 0.01 and ^###^ *p* < 0.001 for genotype comparisons within the same treatment group. pound; *p*< 0.05 for planned comparisons to the corticosterone/vehicle-treated group within genotype and using Student *T*-tests analyses.
