# Supplementary material for: S 47445 Produces Antidepressant- and Anxiolytic-Like Effects through Neurogenesis Dependent and Independent Mechanisms
Source: Front Pharmacol. 2017 Jul 19;8:462. doi: 10.3389/fphar.2017.00462 (PMC5515821; doi:10.3389/fphar.2017.00462)

Supplementary Figure 1

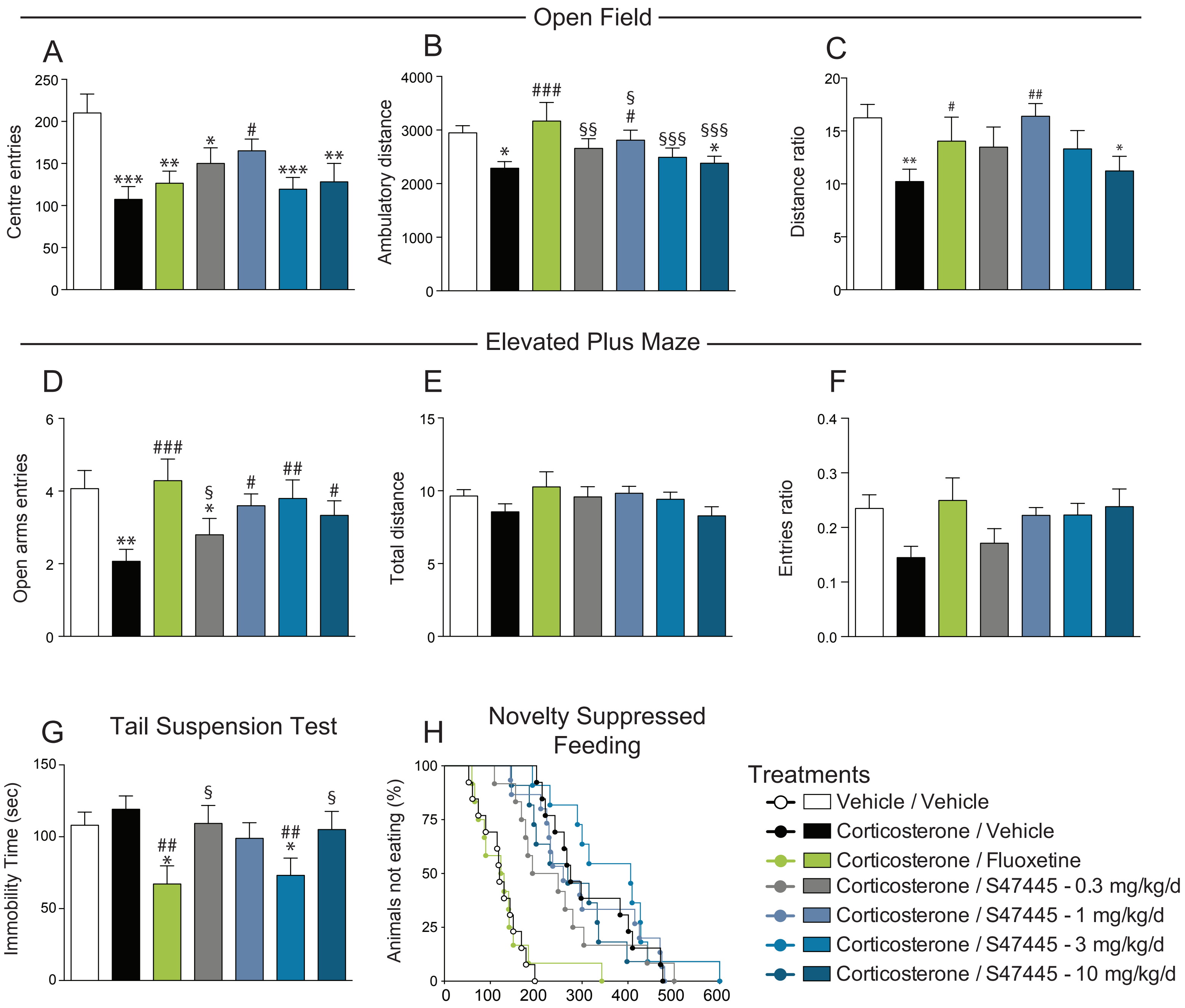

Supplementary Figure 2

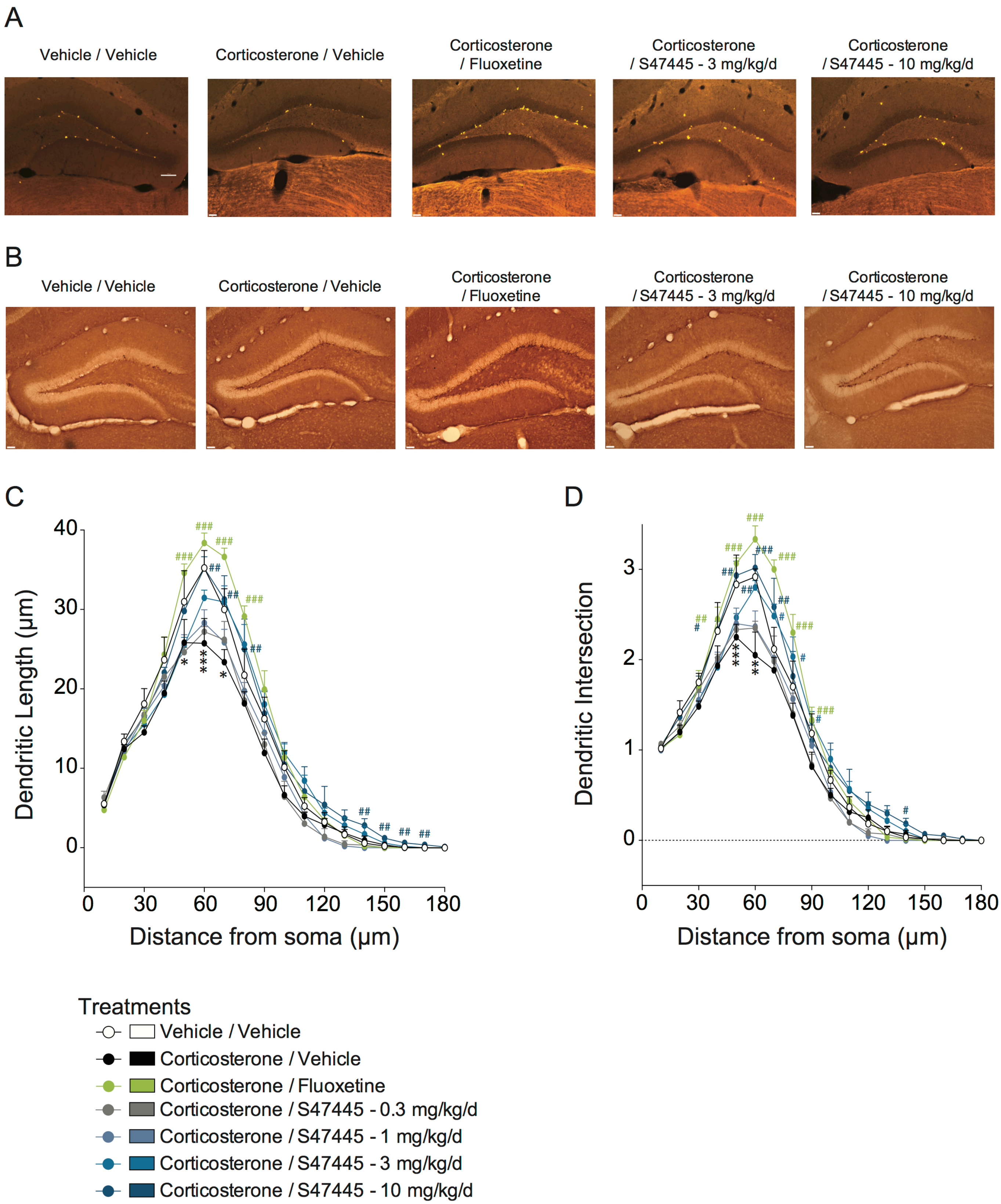

Supplementary Figure 3

A

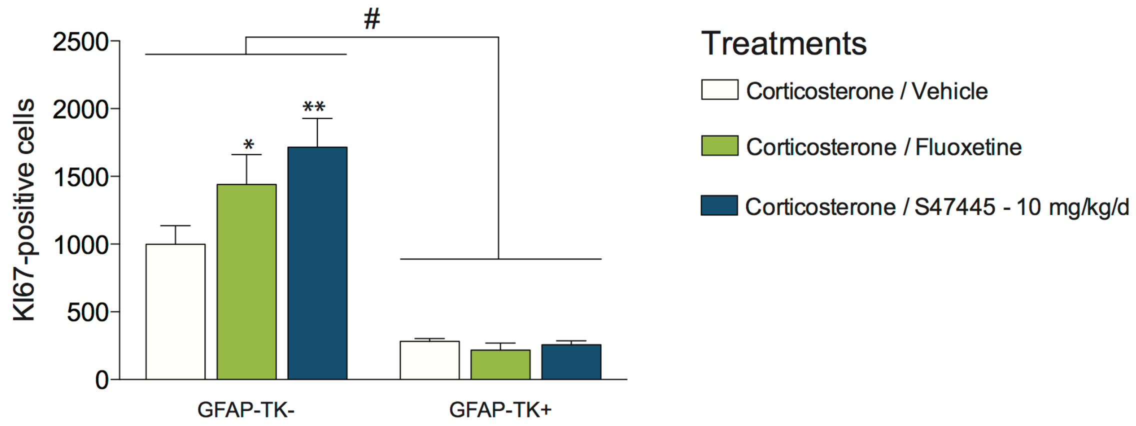

B

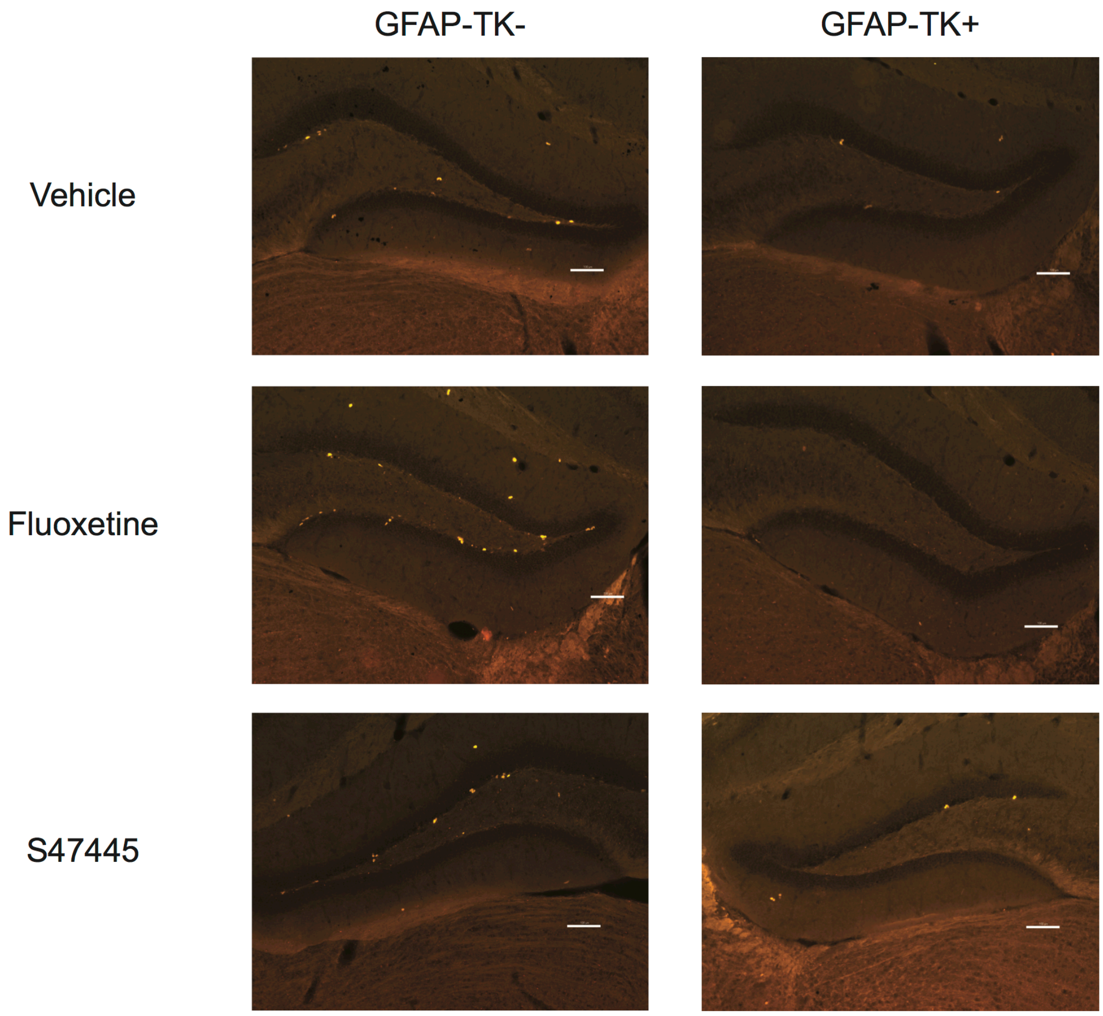

Supplementary Figure 4

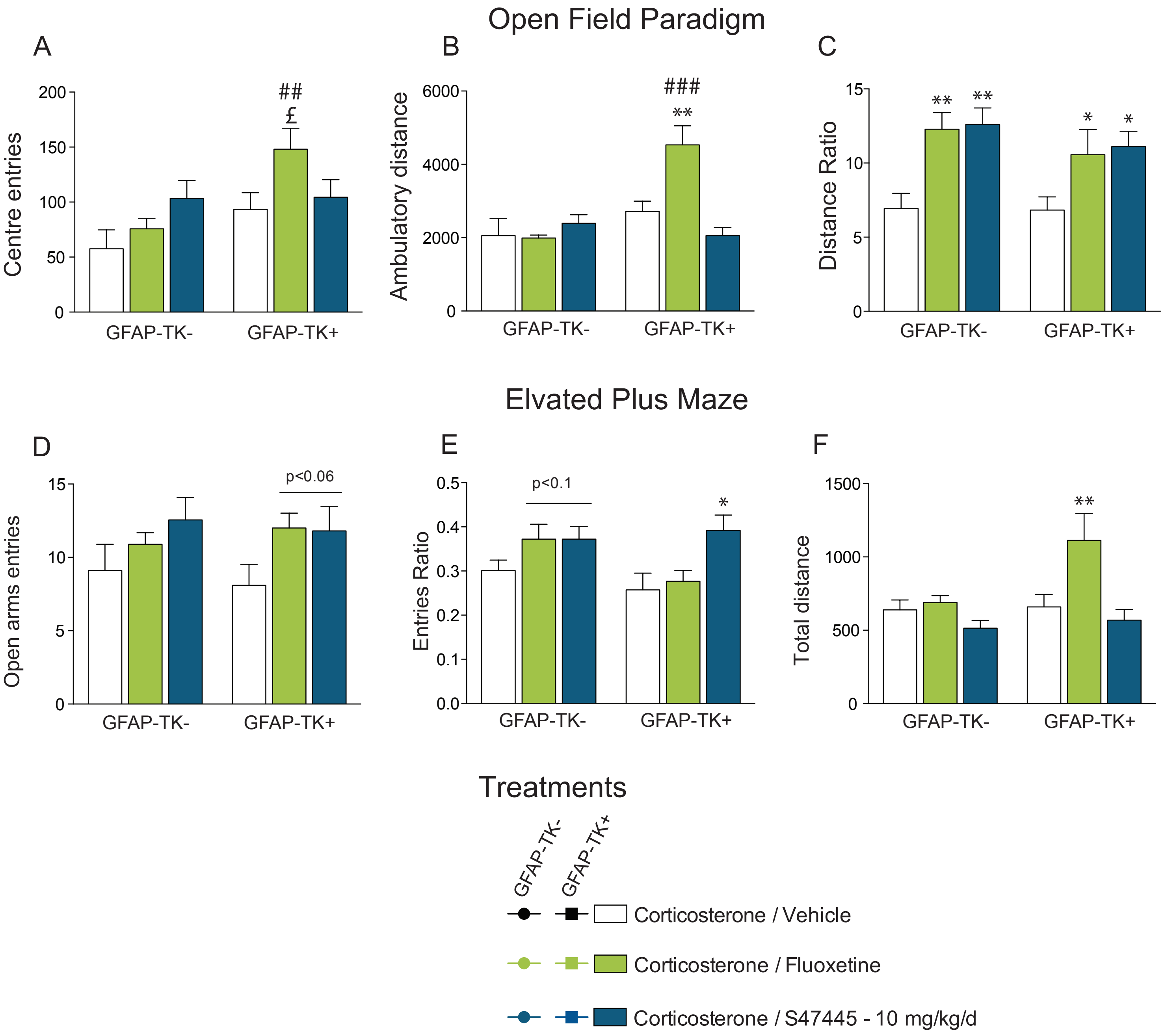

Supplement: FIGURE S1 — Effects of chronic S 47445 or fluoxetine treatment on additional emotionality measurements. Behavioural effects of S 47445 were studied after 28 days of administration at doses of 0.3, 1, 3, and 10 mg/kg, compared to those of vehicle and fluoxetine (18 mg/kg) in corticosterone treated animals. Additional measures of anxiety-related behavior were the number of entries in the centre (A), the ambulatory distance (B), and the distance ratio in the centre of the open-field (C). In the elevated plus maze, the number of entries in open arms (D), the total distance (E), and the entries ratio (F) were recorded. Immobility time in the tail suspension test was measured (G). Data are expressed as mean ± SEM (n = 13–15 animals/group). In the novelty suppressed feeding, latency time to feed was measured and expressed as cumulative survival with percentage of animals that have not eaten over 10 min (H). ∗p< 0.05; ∗∗p< 0.01 and ∗∗∗p < 0.001 for comparisons to the vehicle/vehicle-treated group. #p< 0.05; ##p< 0.01 and ###p < 0.001 for comparisons to the corticosterone/vehicle-treated group. [file Image_1.PDF]
